# Selecting patients with rectal cancer for total neoadjuvant therapy based on clinical lymph node status: do we dare?

**DOI:** 10.2340/1651-226X.2026.45125

**Published:** 2026-02-15

**Authors:** Anna Baech Slipsager, Michael Bødker Lauritzen, Laura Katrine Buskov, Laurids Østergaard Poulsen

**Affiliations:** a Department of Oncology, Aalborg University Hospital, Aalborg, Denmark; b Clinical Cancer Research Center, Aalborg University Hospital, Aalborg, Denmark; c Department of Clinical Medicine, Aalborg University, Aalborg, Denmark; d Department of Gastrointestinal Surgery, Aalborg University Hospital, Aalborg, Denmark; e Department of Radiology, Bispebjerg and Frederiksberg, Copenhagen University Hospital, Copenhagen, Denmark

**Keywords:** rectal neoplasms, neoadjuvant therapy, radiotherapy, risk factors

## Introduction

Total neoadjuvant therapy (TNT) combines neoadjuvant chemotherapy with preoperative (chemo)radiotherapy and offers a significant reduction in the risk of distant recurrence of rectal cancer [1, 2]. According to the European Society for Medical Oncology (ESMO) guideline for localized, lower or middle third rectal cancer, TNT is recommended for patients meeting high-risk criteria: cT4a- or cT4b-stage, involvement of the mesorectal fascia, clinical N2-stage (cN2), extramural venous invasion, and/or lateral lymph node enlargement of ≥ 7 mm [3]. It is essential that these clinical variables exhibit high diagnostic accuracy to justify the use of intensive treatment regimes with potential side effects [4]. A low accuracy increases the likelihood of overtreatment.

Magnetic resonance imaging (MRI) has become the gold standard for local staging of rectal cancer, leading to improvements in diagnostic precision [5]. However, MRI-based lymph node involvement has low sensitivity [6]. Despite this knowledge and lack of evidence regarding diagnostic accuracy, cN2 is recommended as a high-risk criterion.

Applying the ESMO guidelines in the treatment setting necessitates evaluation of the accuracy of regional lymph node staging by MRI in rectal cancer. The aim is to assess, on real-world data, how MRI-based nodal staging corresponds to the pathological assessment in Danish rectal cancer patients.

## Material and methods

This national cohort study followed the STROBE guidelines [7].

### The European Society of Gastrointestinal and Abdominal Radiology guidelines

The European Society of Gastrointestinal and Abdominal Radiology (ESGAR) guidelines from 2016 have been applied for staging rectal cancer since 2017. The guideline’s criteria for suspicious regional lymph nodes include either a short axis diameter of ≥ 9 mm, a short axis diameter of 5–8 mm and ≥ 2 malignant characteristics, or a short axis diameter of < 5 mm and three malignant characteristics. The malignant characteristics are round shape, heterogeneous appearance, and spiculated or indistinct borders. All mucinous lymph nodes are considered malignant [8]. MRI-based cN-staging is determined by number of suspicious lymph nodes. cN0-stage is no suspicious lymph nodes, cN1-stage is 1–3 suspicious lymph nodes, and cN2-stage is 4 or more suspicious lymph nodes. When lymph node status is not evaluated, Nx-stage is noted [9].

### The TNM pathological classification

The TNM Pathological Classification is used to declare the number of regional lymph nodes with malignant cells. pN0-stage is no spread of malignancy to the lymph nodes, pN1-stage is spread to 1–3 lymph nodes, and pN2-stage is spread to 4 or more lymph nodes. When the lymph node status is not evaluated, pNx-stage is noted. A minimum of 12 lymph nodes must be assessed to reduce the risk of understaging lymph node involvement [10].

### Patient cohort

The Danish Colorectal Cancer Group database registers Danish rectal cancer patients with an overall accuracy of 95% [11]. Patients with rectal cancer diagnosed between January 1, 2020 and June 26, 2025 who underwent high-resolution MRI, multidisciplinary team discussion, and surgery were identified from the database. Exclusion criteria were preoperative oncological treatment, unknown number or less than 12 lymph nodes in the surgical specimen, synchronous colorectal cancer, metastatic disease at time of diagnosis, an MRI-diagnosed and/or pathological N-stage registered as missing data, and a MRI-diagnosed and/or pathological T-stage registered as T0-stage or missing data. Risk factors were assessed only in patients with cN2-stage and defined as cT4-stage, tumor deposit, and/or < 1 mm distance to the mesorectal fascia (cT3 tumors only). Missing data were classified as no risk factor.

### Statistical analysis

Descriptive statistics were used to summarize baseline characteristics. The accuracy of MRI-based lymph node status was analyzed by comparison with the pathological nodal stage using a frequency table. Results were presented as percentages with 95% confidence intervals using the exact binomial method (Clopper–Pearson). The associations between accuracy of MRI-based lymph node status and sex as well as tumor level were evaluated using chi-square test. A *p*-value < 0.05 was considered statistically significant. Statistical analyses were performed using R software [12].

## Results

**Figure 1:**
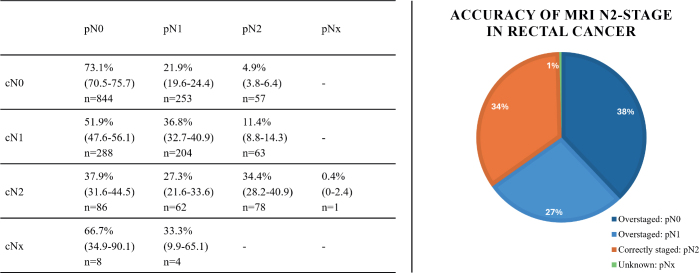
Comparison of MRI-based N-stage (cN) with pathological N-stage (pN). Data presented as percentage (95% CI) and n = number of patients per subgroup. *CI: confidence interval; MRI: magnetic resonance imaging.

In our analysis, 1948 patients were included (Table 1). The median time from diagnosis to surgery was 17 days (interquartile range [IQR]: 12.0, 25.0). Among the 227 patients diagnosed with cN2-stage, 34.4% had pN2-stage disease and 37.9 and 27.3% had pN0-stage and pN1-stage disease, respectively (Figure 1).

**Table 1:** Patient characteristics.

Number of patients	1948
Sex - Female - Male	756 (38.8%) 1192 (61.2%)
Age (median (IQR))	69.00 [60.00, 77.00]
cT-stage - cT1 - cT2 - cT3 - cT4 - cTx	316 (16.2%) 711 (36.5%) 813 (41.7%) 99 (5.1%) 9 (0.5%)
cN-stage - cN0 - cN1 - cN2 - cNx	1154 (59.2%) 555 (28.5%) 227 (11.7%) 12 (0.6%)
pT-stage - pT1 - pT2 - pT3 - pT4 - pTx	308 (15.8%) 637 (32.7%) 902 (46.3%) 97 (5.0%) 4 (0.2%)
pN-stage - pN0 - pN1 - pN2 - pNx	1226 (62.9%) 523 (26.8%) 198 (10.2%) 1 (0.1%)
Time from diagnosis to surgery in days (median (IQR))	17.00 [12.0, 25.0]
Tumor level - Low (0–5 cm) - Medium (>5–10 cm) - High (>10 cm) - Unknown	281 (14.4%) 851 (43.7%) 712 (36.6%) 104 (5.3%)
Number of resected lymph nodes - 12–20 - 21–40 - 41–60 - 61–80 - 81–100 - >100	590 (30.3%) 1050 (53.9%) 252 (12.9%) 51 (2.6%) 4 (0.2%) 1 (0.1%)
Procedure - Low anterior resection - Abdominoperineal Excision - Proctocolectomy - Unknown	1365 (70.1%) 575 (29.5%) 7 (0.4%) 1 (0.0%)

IQR: interquartile range.

A total of 125 patients out of 227 patients diagnosed with cN2-stage had no concurrent risk factors (cT4-stage, tumor deposit, and a distance less than 1 mm to the mesorectal fascia). Besides cN2-stage, an additional risk factor was present in 82 patients and two risk factors in 20 patients. No data were missing for cT4-stage, whereas data for tumor deposit were missing in 22 patients and mesorectal fascia distance was missing in 12 of 154 patients with cT3-stage. There was no association between accuracy of cN2-stage and sex as well as tumor level (*p*-value: 0.11 and 0.29, respectively).

## Discussion and conclusion

This study found that 34.4% of the patients were correctly staged as cN2, while overstaging occurred in 65.2% of the patients.

Brown et al. demonstrated an improvement in lymph node staging accuracy when morphological features were added to the standard size criteria [13]. However, lymph node slides were precisely matched with MRI findings, which is not feasible in clinical practice. Furthermore, a retrospective study found no improvement in nodal staging accuracy and suggested a risk of overtreatment due to overstaging [14]. In general, it can be questioned whether a comparison between radiological and pathological lymph node assessments is reasonable. This is due to the substantial differences in how microscopic nodal metastases, undetectable on MRI, and large reactive benign nodes, visible on MRI, are evaluated [15]. In a study on 2178 patients with rectal cancer, a sensitivity of 38% was identified for clinical lymph node staging, categorized as lymph node negative or positive, compared to the pathological assessment for patients with rectal cancer [6]. Other studies have also documented increased risk of overstaging [16, 17].

In a retrospective study, the increased risk of overtreating based on cN- and cT-stage compared to MRI-based tumor deposit, extramural venous invasion, and mesorectal fascia involvement was supported [18]. It is doubtful that cN2 is useable as a risk factor for treatment selection. Furthermore, the importance of mesorectal lymph node metastasis is questionable, if the rectal cancer does not involve the mesorectal fascia and the total mesorectal excision is performed correctly [19]. Involvement of the mesorectal fascia is associated with increased risk of distant recurrence [20].

The risk factors from ESMOs guidelines for use of TNT are originally defined in the RAPIDO trial, which is one of the pioneer studies regarding TNT [3]. In RAPIDO, the patients in the experimental group versus the standard of care group had 65 and 66% of cN2-stage, 32 and 30% of cT4-stage, 32 and 28% of extramural vascular invasion, 62 and 60% of mesorectal fascia involvement, and 14 and 15% of enlarged lateral lymph nodes, respectively [1]. The number of patients being cN2-staged combined with other risk factors is not described. This makes it difficult to determine cN2 as a high-risk factor for recurrence. Furthermore, the definition of a metastatic lymph node in RAPIDO varies from the ESGAR guideline. In RAPIDO, lymph nodes with a short axis < 5 mm were not considered as metastatic, thereby decreasing the risk of overstaging compared to our study.

In this study, low, mid, and high rectal cancer are included. ESMO guidelines defining cN2 as a high-risk criterion for selection of patients for TNT are for localized rectal cancer in the lower or middle third of the rectum [3]. However, inclusion of low, mid, and high-level tumors should not affect the results regarding accuracy of cN2 compared to pN2 [21]. It is a study limitation that patients did not receive preoperative therapy. This leads to a selection of patients with less advanced rectal cancer, where the enlarged lymph nodes are more likely to be inflammatory compared to enlarged lymph nodes in more advanced rectal cancer [22]. This could falsely decrease the sensitivity of the cN2-stage. There were no standardized criteria of the time interval between diagnosis and surgical intervention. The median time to surgery was 17 days. Therefore, the risk of higher pN-stage due to tumor growth is considered low. It was not possible to verify strict use of the 2016 ESGAR guideline by radiologists, though applied in Danish guidelines. As a national study, a strength is a complete and large patient cohort based on high-quality data [11]. The overall patient cohort was rather large (*n* = 1948), but only 227 patients were staged as cN2-stage, which is a limitation due to the relatively small number of patients in this subgroup.

If the results of the present study are applied to the ESMO guidelines for TNT [3], potentially two-thirds of the patients with MRI-based cN2-stage would be overstaged and consequently treated with TNT. The risk of serious adverse events is higher with TNT than with standard neoadjuvant chemoradiotherapy [4]. These patients might be exposed to unnecessary side effects. The standard treatment with neoadjuvant (chemo)radiotherapy is sufficient for most patients with high-risk criteria [23]. When cN2-stage is the only risk factor, it appears to be too inaccurate as a stand-alone criterion in selecting rectal cancer patients benefitting from TNT.

## Data Availability

Data are not assessable due to the Danish General Data Protection Regulation.
